# Little Busy Bee

**Published:** 1887-08

**Authors:** Howard DeTravers


### LITTLE BUSY BEE.

Hilda, mind ! that bee will sting you !
Ouch ! you saucy little thing you ;
Are the garden roses few,
That you're wanting Hilda's too ?
Only hear him where he goes,
Buzzing all about your nose !
Hilda, smiling, answered then,
"*Bees are not as shy as men!*"

Happy bee, I said, that sips
Sweets of Hilda's rosy lips.
Thing so bold, in all my wooing,
I had never ventured doing.—
Hilda tossed her dainty head,
"Stupid !" that was all she said,
But I wonder now, if she
*Really meant it for the bee.*

—HOWARD DETRAVERS.

